# The Thief in the Mirror

**DOI:** 10.1371/journal.pbio.0060201

**Published:** 2008-08-19

**Authors:** Frans B. M de Waal

## Abstract

The few animals capable of recognizing themselves in a mirror have advanced social cognition related to adopting the perspective of someone else.

The Eurasian magpie (Pica pica) has a poor reputation. As a child, I learned never to leave small shiny objects, such as teaspoons, unattended outdoors as these raucous birds will steal anything they can put their beaks on. This folklore even inspired a Rossini opera, “*La gazza ladra*” (“The Thieving Magpie”). Nowadays, this view has been replaced with one that is more sensitive to ecological balance, in which magpies are depicted as murderous plunderers of the nests of innocent songbirds. Either way, they are black-and-white gangsters.

But no one has ever accused a magpie of being stupid. The bird belongs to the Corvidae, a worldwide family (also including crows, ravens, jackdaws, jays, and nutcrackers) marked by an exceptionally large forebrain, which permits innovative foraging [1]. In recent years, this family has begun to pose a challenge to the idea that primates constitute the pinnacle of cognitive evolution by showing creative tool-use, visual perspective-taking, foresight, and so on [2].

The one cognitive accomplishment still missing, and therefore sometimes glossed over by corvid fans, is recognition of oneself in a mirror, a capacity found in only a handful of large-brained mammals. A great deal has been made of this capacity, including turning it into a touchstone for the legal concept of “personhood” [3].

The typical mirror mark test, developed four decades ago by Gordon Gallup [4], seeks to determine whether an animal recognizes itself in the mirror by marking a visible colored dot on the animal's body. The mark needs to be placed on an out-of-view body part so that it can be detected only with guidance of a mirror. The mark test determines if the animal can use its reflection to locate the mark on its body, as measured by its inspection, touching, or rubbing of the spot. Obviously, an animal that considers its mirror image as another individual should perceive the mark as the other's problem, not its own. Very few species pass the mark test of mirror self-recognition (MSR), despite hundreds that have been tested. Apart from humans, strong indications for MSR have been obtained only for the four great apes, bottlenose dolphins, and Asian elephants [5–7].

MSR is sometimes seen as a Rubicon, setting animals with a sense of self apart from those lacking such a sense. This dividing line has been questioned in several ways. Behaviorists, for example, have tried to reduce MSR to conditioning, claiming that the relationship between self and mirror can be learned. They successfully trained pigeons to peck at dots on themselves in front of a mirror [8], but another team that tried to replicate this feat never managed to do so, resulting in a paper with the word “Pinocchio” in its title [9].

These pigeon experiments are irrelevant to Gallup's mark test, however, since the critical issue is whether animals spontaneously connect their reflection with their own body. Training the target behavior drains the mark test of its meaning. No untrained pigeon has ever managed to use a mirror to find dots on its own body.

Other critics of the Rubicon have been more subtle by either making the point that, obviously, all animals must have a sense of self, because otherwise how could they possibly navigate their environment, or by questioning the mark test as the best or sole criterion [10–12]. But for better or worse, this test has remained the gold standard of self-identity.

The new magpie study published in this issue of *PLoS Biology* by Helmut Prior and co-workers [13] of the Ruhr-University in Bochum, Germany, is as well-controlled as any mirror study on other self-recognizing species, and in fact better controlled than most ape and human child studies, which generally fail to include “sham” marks. A sham mark is applied in the same way as a visible mark, and supposed to feel and smell the same, but cannot be visibly detected. In the magpie study [13], this was done by placing a black mark onto the magpies' black throat feathers.

Placed on the same black throat feathers, the visible mark—a tiny colored sticker—stood out, but only in a mirror. Put in front of a mirror, the magpies kept scratching with their foot until the mark was gone, whereas they left the sham mark alone. They also didn't do the same amount of frantic scratching if there was no mirror to see themselves in. Evidently, their self-preening was guided by visual feedback from the mirror.

From an evolutionary perspective, it must be added that MSR seems hardly interesting. It cannot be an important adaptation, since animals lacking this capacity have no trouble with reflective surfaces, such as standing pools of water. Animals certainly do not need to recognize themselves to survive. The importance of the mirror test rather resides in what it may tell us about how animals perceive themselves in relation to their environment, including their social partners. In other words, the mirror test is interesting not because it shows that an animal has the capacity for self-recognition but because of the cognitive abilities that are associated with MSR.

It has been speculated that MSR coincides with advanced social relationships, including the capacity to look at the world from another's viewpoint. Gallup [14] already speculated about this connection, and more recently this idea has been connected to the various levels of empathy reached by mammals. The higher levels of empathy require individuals to grasp the situation in which another finds itself, hence looking at the situation from another's perspective. The same capacity may be reflected in MSR. This is known as the “co-emergence hypothesis,” according to which the capacities for MSR and perspective-taking appear in tandem during both evolution and development [15].

With regards to human development, this hypothesis is well-supported. Children begin to show perspective-taking abilities at around the same time that they first pass the mirror mark test, even after age has been controlled for [16,17]. In the future, researchers may be able to address this issue more directly through neural investigation. In humans, for example, the right inferior parietal cortex, at the temporoparietal junction, underpins advanced empathy by helping distinguish between self- and other-produced actions [18]. If mirror responses tap into the same self–other distinction, the mark test is obviously more than it appears.

Where do magpies fit into this larger scheme? Perhaps perspective taking is critically important for a species that plunders the nests of others and steals from humans. But more likely, this capacity may serve relations among conspecifics. Magpies occasionally cache food and may raid each others' caches, the way their close relatives, the jays, do. For scrub jays, there is evidence that “it takes a thief to know a thief,” suggesting that these birds extrapolate from their own pilfering experiences to the intentions of others [19]. Even if their perspective-taking is not used to assist others, but rather to outwit them, the same grasp of another's situation may be needed as in advanced empathy, perhaps requiring the same parsing of self from other.

Although there remain many unknowns in the study of self awareness, establishing a connection with social cognition seems a promising angle, which may well extend to the magpie. As the ultimate bird thief, this species may have more need than most to guess the intentions of others.

Whatever our conclusions, at the very least the self-recognition of *La gazza ladra* offers fresh meaning to its love of reflective items.
